# Mitochondrion-Targeted Peptide SS-31 Inhibited Oxidized Low-Density Lipoproteins-Induced Foam Cell Formation through both ROS Scavenging and Inhibition of Cholesterol Influx in RAW264.7 Cells

**DOI:** 10.3390/molecules201219764

**Published:** 2015-12-01

**Authors:** Shuangying Hao, Jiajie Ji, Hongting Zhao, Longcheng Shang, Jing Wu, Huihui Li, Tong Qiao, Kuanyu Li

**Affiliations:** 1 Jiangsu Key Laboratory for Molecular Medicine, Medical School of Nanjing University, Nanjing 210093, China; shuangying9088@163.com (S.H.); zhting1210@163.com (H.Z.); sjbslc@126.com (L.S.); wujinghappy@126.com (J.W.); hhli900217@163.com (H.L.); 2 Department of Vascular Surgery, The Affiliated Drum Tower Hospital of Nanjing University Medical School, Nanjing 210008, China; jijiajie071@foxmail.com

**Keywords:** SS-31, CD36, lipid accumulation, macrophage, foam cells, oxidative stress, inflammation

## Abstract

Foam cell formation as a result of imbalance of modified cholesterol influx and efflux by macrophages is a key to the occurrence and development of atherosclerosis. Oxidative stress is thought to be involved in the pathogenesis of atherosclerosis. SS-31 is a member of the Szeto-Schiller (SS) peptides shown to specifically target the inner mitochondrial membrane to scavenge reactive oxygen species. In this study, we investigated whether SS-31 may provide protective effect on macrophage from foam cell formation in RAW264.7 cells. The results showed that SS-31 inhibited oxidized low-density lipoproteins (ox-LDL)-induced foam cell formation and cholesterol accumulation, demonstrated by intracellular oil red O staining and measurement of cholesterol content. The mechanism was revealed that SS-31 did not only significantly attenuated ox-LDL-induced generation of reactive oxygen species (ROS) and increased the activities of superoxide dismutases, but also dose-dependently inhibited the expression of CD36 and LOX-1, two scavenger receptors of ox-LDL, while the expression of ATP-binding cassette A1 and G1, playing a pivotal role in cholesterol efflux, was not affected. As a result, SS-31 decreased pro-inflammatory cytokines such as interleukin 6 and tumor necrosis factor alpha, suggesting the prevention of inflammatory responses. In conclusion, our results demonstrate that SS-31 provides a beneficial effect on macrophages from foam cell formation, likely, through both ROS scavenging and inhibition of cholesterol influx. Therefore, SS-31 may potentially be of therapeutic relevance in prevention of human atherogenesis.

## 1. Introduction

Atherosclerosis, which is a leading cause of death in western societies, has now become a worldwide epidemic affecting nearly all developing and developed countries. It is a chronic systemic multifactorial disease affecting the entire arterial tree, associated with oxidative stress [1,2] and impaired inflammatory status [3,4,5]. Atherosclerotic lesion occurrence is determined by foam cell formation, which is associated with increased cholesterol in macrophages [6]. Foam cell formation primarily resulted from imbalance between modified cholesterol influx and efflux.

Macrophages may express a few scavenger receptors, among of which CD36, LOX-1, and class A scavenger receptor are reported to play important roles in the uptake of ox-LDL [7]. CD36 has been identified to recognize not only lipid moieties of ox-LDL and bind and internalize ox-LDL, but also a range of ligands including anionic phospholipids, apoptotic cells, long-chain fatty acids and other modified LDL [8,9,10]. Mutations of LOX-1 gene have been associated with atherosclerosis. Additionally, cholesterol efflux from macrophages is regulated by various factors in which ATP-binding cassette transporters A1 and G1 (ABCA1 and ABCG1) play a pivotal role [7]. ABCA1 plays an important role in the efflux of cellular cholesterol to extracellular acceptors, such as lipid-free or lipid-poor apolipoprotein A-I (ApoA-1) [11]. In contrast, ABCG1 is majorly expressed in macrophages to mediate the efflux of cholesterol to high-density lipoproteins (HDL) [11].

Oxidative stress plays an important role in the pathogenesis of atherosclerosis. Oxidative stress occurs as a result of an imbalance between the production of ROS and the antioxidant systems of a cell, in which case the effectiveness of antioxidant systems decreases or the production of ROS increases. ROS are considered to be implicated in the pathogenesis of virtually each stage of vascular lesion formation in atherosclerosis [1]. Previous studies have demonstrated that use of celastrol could prevent foam cell formation and atherosclerosis *in vitro* and *vivo* [12].

The production of ROS, such as superoxides, hydrogen peroxide, and peroxynitrite, together with inflammatory factors such as cytokines, chemokines, and adhesion molecules, has been shown to be increased in atherosclerotic lesions [3,4,12]. Recent studies have established a basic role for inflammation in mediating the development of this disease from initiation through progression and, ultimately, the thrombotic complexity of atherosclerosis [13,14,15]. These new evidences favor the important links between risk factors and mechanisms of atherogenesis.

SS-31, a new and innovative mitochondrion-targeted antioxidant, has an alternating aromatic-cationic structure that allows it to freely cross the cell membrane and concentrate several hundred folds in the mitochondrial inner membrane independently of mitochondrial membrane potential [16]. SS-31 interacts with mitochondrial cardiolipin [17], improves ATP production, reduces mitochondrial ROS production, and decreases oxidative damage [18]. These effects are associated with protection against ischemia-reperfusion injury [18], amyloid-β toxicity in Alzheimer’s disease neurons [19], cardiac hypertrophy and failure [20], and MPTP-induced dopaminergic neuron cell death, a model of Parkinson’s disease [21] in animal models. However, the anti-oxidative effect of SS-31 on atherosclerosis has not been investigated. Here, we report that treatment with SS-31 significantly inhibits ox-LDL-induced foam cell formation and reduces oxidative stress and inflammation in RAW264.7 cells.

## 2. Results

### 2.1. SS-31 Reduces Ox-LDL-Induced Cholesterol Accumulation in RAW264.7 Cells

**Figure 1:**
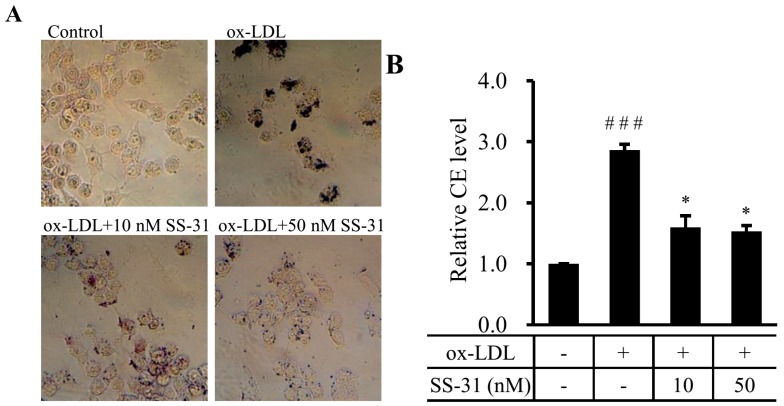
SS-31 reduces ox-LDL-induced lipid accumulation in RAW264.7 cells. (**A**) RAW264.7 cells were exposed to ox-LDL (50 μg/mL) in the presence or absence of SS-31 (10 or 50 nM) for 24 h. Representative photographs showing RAW264.7 cells stained with oil red O. The dark color indicates the stained lipids and/or lipoproteins; (**B**) Levels of CE in RAW264.7 cells. Values represent mean ± SD. Treatment of the cells is the same as in (**A**). Measurement of CE is described in the Experimental Section. Results are quantitative data from four independent experiments. ^###^ *p* < 0.001 compared with the control group, * *p* < 0.05 compared with the ox-LDL group. CE: cholesteryl ester.

The uptake of ox-LDL by macrophage triggers foam cell formation and initiates the development of atherosclerosis. Therefore, we first assessed the effect of SS-31 on foam cell formation in ox-LDL-elicited RAW264.7 macrophages. Oil red O staining and measurement of cholesterol content were performed. Supplementation with ox-LDL to the culture medium induced the foam cell formation as the cytoplasmic lipid droplet accumulation and cellular cholesterol level were apparently increased (Figure 1A), indicating a working cell model of atherosclerosis. Attractively, both ox-LDL induced-lipid droplet accumulation and cellular cholesterol level were markedly decreased by treatment with SS-31 in a dose dependent manner (Figure 1A,B). The results suggest that SS-31 prevents ox-LDL-induced foam cell formation in RAW264.7 cells.

### 2.2. SS-31 Suppresses Ox-LDL-Induced Oxidative Stress in RAW264.7 Cells

**Figure 2:**
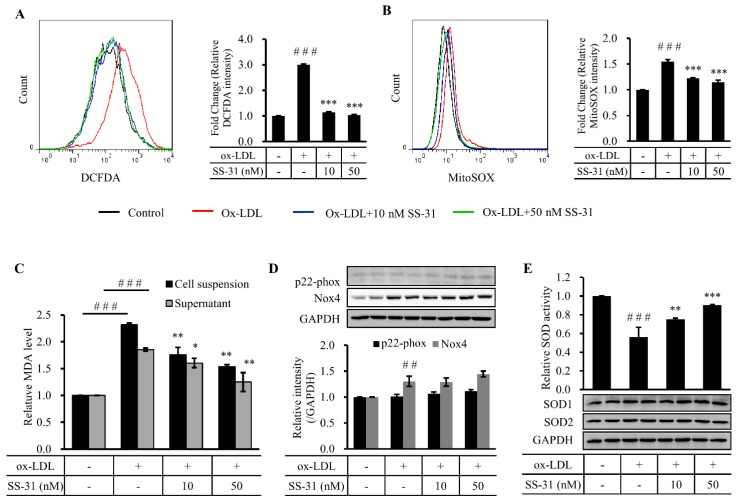
SS-31 suppresses ox-LDL-induced oxidative stress in RAW264.7 cells. (**A**) Representative fluorescence-activated cell sorter (FACS) analysis with a free radical sensor H_2_DCFDA (left panel) and relative mean fluorescence intensity of H_2_DCFDA (right panel) indicates intracellular ROS production. Cells were treated the same as in Figure 1 before treatment of H_2_DCFDA; (**B**) Representative FACS analysis with MitoSOX Red, a mitochondrial O_2_^−^ specific dye, (left panel) and relative mean fluorescence intensity (right panel) indicates mitochondrial ROS production; (**C**) Levels of cell suspension and medium supernatant MDA content; (**D**) A representative Western result (upper panel) and quantitative data (lower panel) of the expression of p22-phox (Nox2) and Nox4 after ox-LDL and/or SS-31 treatment; (**E**) Enzymatic activity of SOD (upper panel in bar graph) and a representative Western blot analysis of SOD1/2 expression (lower panel). GAPDH was used as a loading control. All quantitative data are from four independent experiments. ^###^ *p* < 0.001 compared with the control group, ^##^ *p* < 0.01 compared with the control group. *** *p* < 0.001 compared with the ox-LDL group, ** *p* < 0.01 compared with the ox-LDL group, * *p* < 0.05 compared with the ox-LDL group. ROS: reactive oxidative species; MDA: malondialdehyde; SOD: superoxide dismutase.

Oxidative stress has been implicated in the pathogenesis of various cardiovascular diseases including atherosclerosis [22]. Reduction of oxidative stress by preventing ROS generation and/or accelerating ROS inactivation may represent therapeutic strategies for the treatment of atherosclerosis. To estimate the effect of SS-31 on ox-LDL-induced level of cellular oxidative stress in RAW264.7 cells, we used the two oxidative stress probes DCFDA and mitoSOX, respectively, to analyze cytosolic and mitochondrial ROS production by flow cytometry (Figure 2A,B). The results showed that ROS generation significantly increased after treatment of ox-LDL in both organelles. Co-treatment with SS-31 drastically not only prevented mitochondrial ROS generation, but also reduced cytosolic ROS levels. In consistence with that, ox-LDL markedly increased levels of malondialdehyde (MDA), a biomarker of oxidative stress, in cell suspensions and supernatants, which was inhibited by co-treatment with SS-31 (10 or 50 nM) (Figure 2C). It has been reported that ox-LDL-induced ROS generation is mediated by NADPH oxidase (Nox). Therefore, we measured two of subunits of Nox, Nox2 and Nox4. The level of Nox2 was constant during the whole experiment, while a significant increase of Nox4 after ox-LDL treatment was observed. However, no change of Nox4 was revealed after SS-31 treatment (Figure 2D), suggesting that at least partial ox-LDL-induced ROS production came from Nox4 and the drop of the ROS levels by SS-31 treatment is not because of the less generation of ROS by Nox4. Superoxide dismutase (SOD) catalyzes the dismutation of superoxide into oxygen and hydrogen peroxide thereby serving a key antioxidant function [23]. In the previous study, SOD2 deficiency (SOD2+/−) leads to mitochondrial dysfunction, increased mitochondrial DNA damage and accelerated atherosclerosis in ApoE-KO mice [24]. Here, we measured the protein levels of SOD1/2 and the activity of total SOD. Treatment of RAW264.7 cells with ox-LDL significantly inhibited the activity of SOD, which was recovered in a dose-dependent manner by co-treatment with SS-31 (10 or 50 nM) (Figure 2E). However, this was observed without either concomitant changes of the protein levels of the two superoxide scavenging enzymes SOD1 and SOD2. Altogether, these data suggest the protective effects of SS-31 against ox-LDL-induced oxidative stress through directly scavenging ROS and maintaining the activities of SOD in RAW264.7 cells.

### 2.3. SS-31 Down-Regulates Ox-LDL-Induced Expression of CD36 and LOX-1 in RAW264.7 Cells

**Figure 3:**
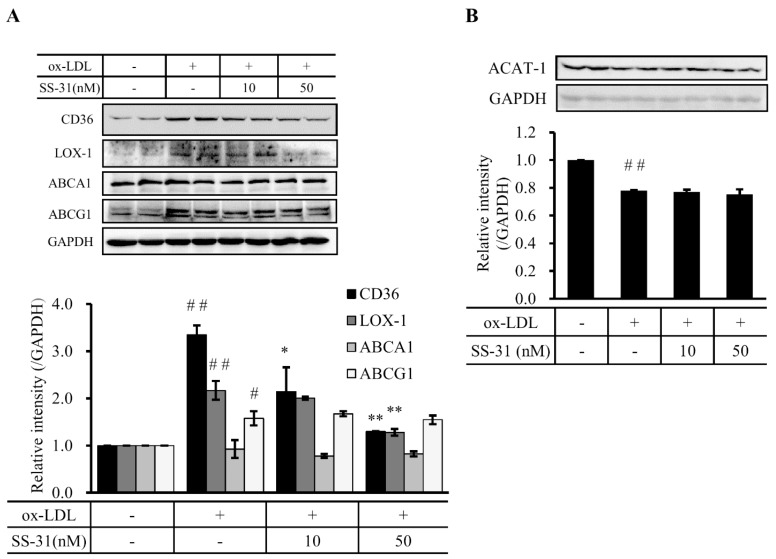
SS-31 down-regulates cholesterol influx in a dose dependent manner, but does not affect cholesterol efflux and endogenous cholesterol biosynthesis. (**A**) A representative western blot result (upper panel) and quantitative data (lower panel) of CD36, LOX-1, ABCA1, and ABCG1 protein levels after ox-LDL and/or SS-31 treatment of RAW264.7 cells; (**B**) A representative western blot result (upper panel) and quantitative data (lower panel) of the protein level of ACAT-1. GAPDH was used as a loading control. Cells were treated the same as in Figure 1. Results are from four independent experiments. ^#^ *p* < 0.05 and ^##^ *p* < 0.01 compared with the control group, * *p* < 0.05 and ** *p* < 0.01 compared with the ox-LDL group.

In view of the fact that foam cell formation primarily resulted from imbalance between cholesterol influx and efflux, we asked whether treatment of SS-31 affected cholesterol trafficking. Therefore, the expression of CD36 and LOX-1, two scavenger receptors of cholesterol influx, and ABCA1 and ABCG1, two important transporters for cholesterol efflux, were detected after SS-31 treatment. Very interestingly, the result showed that ox-LDL-induced upregulation of CD36 and LOX-1 was inhibited in a dose-dependent manner by SS-31 treatment (Figure 3A). However, the expression of ABCA1 and ABCG1 did not change (Figure 3A). These data suggest that SS-31 likely suppresses the uptake of ox-LDL by diminishing the expression of the scavenger receptors CD36 and LOX-1.

Due to the fact that macrophages also make cholesterol which may drive foam cell formation, we evaluated the endogenous cholesterol biosynthesis by tracing the indicator ACAT-1, which is implicated in atherosclerotic plaques by controlling the equilibrium between free cholesterol and cytoplasmic cholesteryl esters. A slight, but significant, decrease of ACAT-1 protein level was observed after ox-LDL treatment in RAW264.7 cells (Figure 3B). Co-culture with SS-31 did no alter this decrease, suggesting that the endogenous cholesterol biosynthesis did not contribute to the foam cell formation of RAW264.7 cells in our test condition.

### 2.4. SS-31 Inhibits Ox-LDL-Induced Inflammatory Response in RAW264.7 Cells

**Figure 4:**
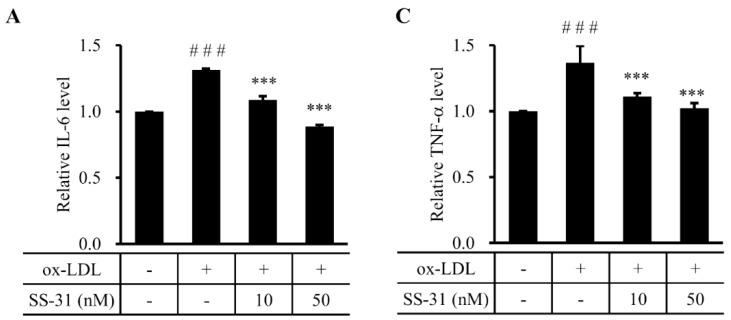

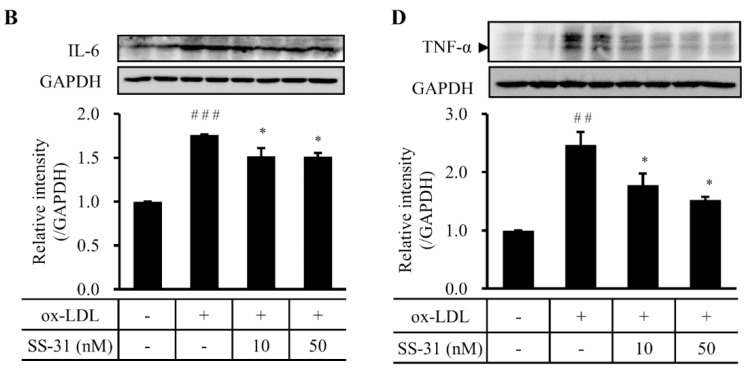
SS-31 inhibits ox-LDL-induced inflammation in RAW264.7 cells. (**A**) ELISA and (**B**) western blot analysis of IL-6 protein; (**C**) ELISA and (**D**) western blot analysis of TNF-α protein. A representative graph of a western blot is shown here for IL-6 and TNF-α, respectively. GAPDH was used as a loading control. The cells were treated the same as in Figure 1. All experiments were repeated four times independently. ^###^ *p* < 0.001 compared with the control group, * *p* < 0.05 and *** *p* < 0.001 compared with the ox-LDL group.

To verify the effect of SS-31 on ox-LDL-induced foam cell formation, ELISA and western blot analysis were performed to detect the inflammatory cytokine production in RAW264.7 cells. The results showed that ox-LDL-induced increase of interleukin 6 (IL-6) (Figure 4A,B) and tumor necrosis factor alpha (TNF-α) (Figure 4C,D) levels were significantly reduced by treatment of SS-31, suggesting that SS-31 may efficiently prevent from the ox-LDL-induced occurrence of inflammation.

## 3. Discussion

**Figure 5:**
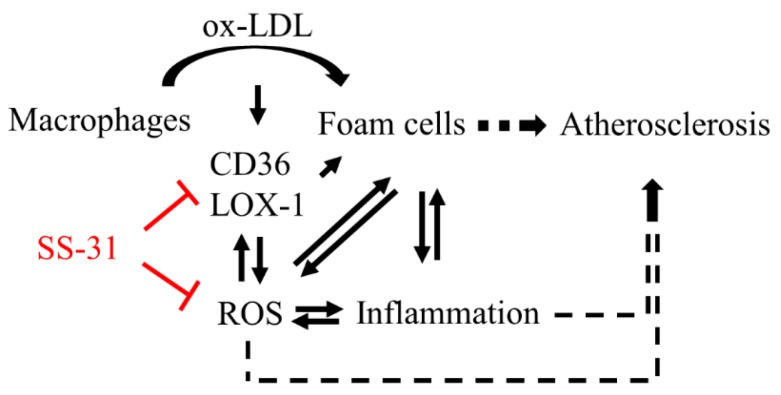
Schematic diagram for suppression of ox-LDL-elicited foam cell formation by SS-31. Ox-LDL stimulates its own uptake by induction of macrophages surface receptor CD36 and LOX-1 expression, resulting in the increased influx of ox-LDL to promote macrophages converting into foam cells. Foam cell formation triggers generation of ROS, which interplays with inflammation. SS-31 reduces production of ROS and prevents the upregulation of CD36 and LOX-1, consequently decreases foam cell formation.

Atherosclerosis is a chronic disease characterized by the accumulation of excessive cholesterol in the arterial intima. Macrophage foam cell formation is critical in the occurrence and development of atherosclerosis. In this study, we demonstrate that SS-31 may efficiently prevent from macrophage foam cell formation. Not only could SS-31 inhibit ROS generation and inflammatory response, two synergic activities on atherogenesis, but also directly prevent the cholesterol uptake, summarized in Figure 5. The effects of SS-31 in the murine macrophage cell line RAW264.7 as a model of atherosclerosis is very interesting and promising for predicting the benefits of therapy for this compound.

Szeto and Schiller developed a mitochondrion-targeted polypeptide (SS-31) with antioxidant action [16], which has been verified in number of cell/animal models of human diseases [17,18,19,20]. Based on these very interesting and promising preclinical data, the SS peptides have entered into phase II clinical development [25,26]. The mechanism is supposed to be that SS-31, by interacting with cardiolipin, prevents cardiolipin from converting cytochrome *c* into a peroxidase while protecting its electron carrying function [27] to protect mitochondrial integrity and prevent ROS generation. Oxidative stress accelerating foam cell formation is of fundamental importance for atherosclerosis. Many antioxidants such as vitamin E, Didox, mitoQ and celastrol had been validated to ameliorate atherosclerosis *in vivo* and/or *in vitro* [12,28,29,30]. Although not yet are the clinical trials with these reagents for atherosclerosis, our preclinical data support the protective effects of SS-31 from foam cell formation in the cell model. The *in vivo* study will be conducted in the near future to explore the potential benefits of therapy for this compound in an animal model of atherosclerosis.

We measured both supernatant and cellular changes of MDA levels, which reflected lipid peroxidation. Biological membranes contain a high content of polyunsaturated fatty acid, so they are particularly susceptible to peroxidative attack. The decrease of cellular and supernatant MDA levels by co-treatment of SS-31 suggests less ROS generation and lipid peroxidation, therefore less diffused MDA from cytoplasm to extracellular environment. The antioxidant activity of SS-31 unlikely occurs in extracellular environment because SS-31 is thought to freely cross the cell membrane and enrich several hundred folds in the mitochondrial inner membrane.

CD36, a class B scavenger receptor, and LOX-1, belonging to the C-type lectin superfamily, are macrophage receptors for ox-LDL and are critical molecules in atherosclerotic foam cell formation. They, as well as scavenger receptor class A, are responsible for uptake of majority of lipoprotein-derived cholesterol in macrophages. Murine atherosclerosis model, crossing the CD36-null mouse into an apolipoprotein E-null atherogenic strain developed significantly less atherosclerosis [31]. Previous studies have also demonstrated that ox-LDL can stimulate its own uptake by induction of CD36 gene expression [32,33]. The mechanism by which ox-LDL upregulating CD36 involves activation of the transcription factor peroxisome proliferator activated receptor gamma (PPAR-γ) [31]. Oxidized LDL have two oxidized linoleic acid metabolites, 9-HODE and 13-HODE, which were PPAR-γ ligands. They induce further CD36 expression via PPAR-γ-responsive elements in the CD36 promoter in monocyte/macrophages [33]. This feedback accelerates macrophages to convert into foam cells. Meanwhile, macrophage lipid peroxidation can be induced by ox-LDL or oxidative stress, and this lipid peroxidation can subsequently stimulates CD36 expression [34,35], which worsens the case. In our study, co-treatment of ox-LDL and SS-31 downregulated the expression of CD36, suggesting the blockage of ox-LDL uptake. On the other hand, macrophage cholesterol efflux protects cells from free cholesterol- and oxysterol-induced toxicity and is mediated via the ABC transporters ABCA1 and ABCG1 [11]. Here we did not find the response of ABCA1 and ABCG to co-treatment of ox-LDL and SS-31. Thus, SS-31 protected macrophage from foam cell formation through inhibition of cholesterol influx rather than promotion of cholesterol efflux to alleviate the lipid accumulation in ox-LDL-elicited macrophages.

As previously reported, IL-6 and TNF-α of the inflammatory mediators, increase intracellular ox-LDL accumulation in THP-1 cells and macrophages in a concentration-dependent manner, whereas administration of IL-6 and TNF-α antibodies may inhibit intracellular ox-LDL accumulation [36]. Blockage of IL-6 and TNF-α may also inhibit ox-LDL-induced production of monocyte chemoattractant protein-1 (MCP-1), a crucial protein in the migration of monocytes into the vessel wall intima where monocytes are transformed into foam cells [37]. Therefore, both IL-6 and TNF-α are considered not only as the markers of inflammatory status but also as stimulators of foam cell formation. In our study, SS-31 treatment reduced the levels of both TNF-α and IL-6, which condition protected cells from inflammatory injury and correlated very well with less foam cell formation.

In summary, the present results demonstrate that treatment with SS-31 significantly inhibited the generation of ROS and promoted the activity of superoxide dismutases. More importantly, SS-31 decreased ox-LDL-induced cholesterol accumulation, inflammation, and foam cell formation via down-regulation of cholesterol influx in RAW264.7 cells. Therefore, SS-31 might potentially be of therapeutic relevance in prevention of human atherosclerosis.

## 4. Experimental Section

### 4.1. Cell Culture and Treatment

RAW264.7 cells were cultured in medium RPMI 1640 with 10% FBS, penicillin (100 U/mL) and streptomycin (100 mg/mL) at 37 °C in a 5% CO_2_ tissue culture incubator. Confluent cells (85%–90%) were incubated with ox-LDL (50 μg/mL, Sigma-Aldrich, St. Louis, MO, USA) in the presence or absence of SS-31 (10 or 50 nM, China Peptides Co. Ltd., Shanghai, China) for 24 h.

### 4.2. Cholesterol Content

The content of lipids including total cholesterol (TC) and free cholesterol (FC) of ox-LDL-treated RAW264.7 cells were measured by enzymatic assay kits according to the protocol from the manufacturer (Abcam, Cambridge, MA, USA). The conjugated cholesterol is calculated as cholesteryl ester (CE) with the following formula: CE = TC − FC.

### 4.3. Oil Red O Staining

For oil red O staining, cells were seeded on 12-well plates and exposed to ox-LDL (50 μg/mL) in the presence or absence of SS-31 (10 or 50 nM) for 24 h. After the previous treatment, cells were fixed in 4% paraformaldehyde (10 min), washed with distilled water, and dried completely before applying 0.6% filtered oil red O solution in 60:40 (*v*/*v*) isopropyl alcohol: H_2_O (room temperature, 30 min).

### 4.4. Western Blot Analysis

Western blot assays were conducted as previously described [38]. Briefly, cells were harvested and completely homogenized using lysis buffer (Thermo Fisher Scientific, Waltham, UK), then were placed on ice for 10 min. After centrifugation at 12,000 rpm for 10 min at 4 °C, the supernatant was collected. Protein concentration was determined with a BCA kit (Beyotime Biotech., Shanghai, China). Proteins (35 μg) were denatured at 95 °C for 5 min in SDS and β-mercaptoethanol-containing sample buffer. The samples were subjected to electrophoresis on 10% or 12% SDS–polyacrylamide gels for 30 min at 80 V followed by 100 min at 100 V and then transferred onto nitrocellulose membrane (PALL, New York, NY, USA) sheets for 90 min at 250 mA. After blocked with 5% skim milk for 90 min at room temperature, the blots were incubated at 4 °C overnight with primary antibodies against CD36 (1:1000 dilution, Proteintech Group, Inc., Chicago, IN, USA), SOD1 and SOD2 (1:1000, Abcam), TNF-α (1:200, Santa Cruz Biotech., Santa Cruz, CA, USA), ABCA1 (1:500, Signalway Antibody LLC, College Park, MD, USA), ABCG1 (1:1000, Proteintech Group, Inc.), ACAT-1 (1:1000, Proteintech group, Inc.), LOX-1 (1:1000, Proteintech group, Inc.), IL-6 (1:1000, Proteintech Group, Inc.), p22-phox (1:200, Santa Cruz Biotech.), Nox4 (1:1000, Abcam), and GAPDH (1:1000, Bioworld Tech., St. Louis Park, MN, USA) as needed. Then the blots were incubated with HRP-conjugated secondary antibodies (goat anti-rabbit or mouse). Blotted-protein bands were visualized with enhanced chemiluminescence detection reagents (Thermo Fisher Scientific). Relative changes in protein expression were estimated from the mean pixel density using Image J, normalized to GAPDH. The levels of IL-6 and TNF-α were also analyzed with ELISA kit (Elabscience Biotech. Co., Ltd., Wuhan, China) according to the manufacturer’s protocol.

### 4.5. Measurement of Cellular ROS Production, SOD Activity and MDA Level

Both intracellular and mitochondrial ROS levels were estimated using a fluorescence-labeled probe H2DCFDA (Beyotime Biotech.) and mitochondrial superoxide indicator MitoSOX Red (Thermo Fisher Scientific), respectively, with flow cytometry analysis. Cellular SOD activity was analyzed with a SOD assay kit and cellular and medium MDA levels with a MDA assay kit following the manufacturer’s protocols, respectively (Nanjing Jiancheng Bioengineering Institute, Nanjing, China).

### 4.6. Statistics Analysis

The values were expressed as mean ± SD. A one-way analysis of variance (ANOVA) was done using SPSS ver. 22.0 software (IBM Corporation, Armonk, NY, USA). Significance was considered at *p* < 0.05.
